# Integrated Multi-Omics Analysis Reveals the Regulatory Mechanism of Peanut Skin Procyanidins on Lipid Metabolism in High-Fat-Diet-Induced Obese Mice

**DOI:** 10.3390/nu17132228

**Published:** 2025-07-05

**Authors:** Jinxin Shen, Yi Zhou, Daijun Yang, Ruonan Liu, Xiaoling Zhu, Rui Liu

**Affiliations:** 1 College of Food Science and Technology, Huazhong Agricultural University, Wuhan 430070, China; 18162193983@163.com (J.S.);; 2 Hubei Provincial Institute for Food Supervision and Test, Wuhan 430075, China

**Keywords:** peanut skin procyanidins, high-fat diet, lipid metabolism, network pharmacology, metabolomics, intestinal microbiota

## Abstract

**Background**: Obesity-associated metabolic disorders represent a critical global health challenge, which necessitates innovative strategies targeting lipid metabolism. Peanut skin procyanidins (PSPs), abundant bioactive compounds derived from agricultural by-products, show potential in lipid regulation, but molecular mechanisms remain unclear. **Methods**: This study integrated hepatic metabolomics, network pharmacology, and gut microbiota analysis to systematically decipher the mechanisms for PSP to ameliorate high-fat diet (HFD)-induced lipid metabolism disorders. **Results**: PSP intervention significantly attenuated HFD-induced increases in LDL-C, TG, and TC levels and effectively mitigated hepatic lipid accumulation. Metabolomics revealed that PSP reshaped hepatic lipid dynamics by modulating glycerophospholipid, linoleic acid, arachidonic acid, tryptophan, and nitrogen metabolism. Subsequent network pharmacology identified *PLA2G10*, *PLA2G5*, *PLA2G2A*, and *CYP1B1* as the core targets, and PSP could markedly suppress their HFD-induced overexpression. Furthermore, PSP selectively reshaped the gut microbiota, enriching beneficial genera such as *Akkermansia* and *Bacteroides* while reducing the abundance of harmful bacteria within Firmicutes. PICRUSt-based functional prediction indicated that PSP alters gut microbial glutamine synthetase activity. **Conclusions**: Mechanistically, PSP regulates lipid metabolism by downregulating *PLA2G10*, *PLA2G5*, *PLA2G2A*, and *CYP1B1* expression, remodeling gut microbiota structure, and increasing hepatic glutamine level. These findings provide novel insights into value-added utilization of agricultural byproducts and development of targeted intervention strategies for metabolic diseases.

## 1. Introduction

Obesity and the associated metabolic syndrome have emerged as a global public health crisis. Epidemiological data have revealed that the obesity rate has reached about 20% among Chinese children and adolescents, which is strongly correlated with chronic conditions such as diabetes and cardiovascular diseases [1,2]. Dysregulated lipid metabolism, which is characterized by impaired lipid absorption, transport, and oxidative balance, is a central mechanism in obesity pathogenesis [1,3]. Recent research has identified critical regulatory targets in lipid metabolism, including the AMP-activated protein kinase (AMPK) signaling pathway (modulating energy homeostasis), peroxisome proliferator-activated receptor gamma (PPARγ) (mediating adipocyte differentiation), and sterol regulatory element-binding protein 1c (SREBP-1c) (promoting fatty acid synthesis) [4,5]. Although dietary intervention is widely recognized as an effective strategy, it remains challenging to precisely target the metabolic pathways through functional components in natural products.

Dietary polyphenols, particularly procyanidins, have attracted significant attention for their excellent bioactive properties. Procyanidins are abundant in fruits and nuts [6], exhibiting the potential to regulate energy homeostasis and suppress adipogenesis [7,8,9,10]. Notably, peanut (*Arachis hypogaea* L.) is an agricultural crop with an annual yield exceeding 19 million tons in China [11], and approximately 500,000 tons of peanut skins are generated as by-products each year. Peanut skins are extremely rich in procyanidins, which account for up to 15% of their dry weight [12], underscoring its potential for value-added utilization. It has been demonstrated that peanut-skin-derived procyanidins (PSPs) can alleviate lipid metabolic disorders through mechanisms such as gut microbiota modulation and AKT signaling inhibition [13,14,15]. However, the regulatory mechanisms of PSP on obesity-associated lipid metabolism remain incompletely elucidated.

The liver is the central hub for lipid metabolism, orchestrating the metabolism of lipids, glucose, and amino acids [16]. Metabolomics allows for the systematic profiling of dynamic lipid metabolite reprogramming and pinpointing the key metabolic nodes under pathological conditions [17]. For example, Laia et al. [18] identified significant differences in the lipid metabolites among obese individuals through lipidomics. Network pharmacology constructs “compound–target–pathway” networks to predict the targets of bioactive components and the associated pathways [19,20]. For instance, Tai et al. [19] revealed which Chinese traditional medicines exert anti-obesity effects by network pharmacology. Network pharmacology on the basis of metabolomics can identify disease-specific targets and signaling pathways, enabling a systematic research workflow from phenotypic metabolic alterations to molecular mechanisms. It establishes a hierarchical framework of “metabolic phenotype → pathway perturbation → target prediction → mechanistic validation.” For instance, in a study exploring the mechanism for quercetin to treat inflammatory lung injury, metabolomics was used to identify the dysregulated metabolic pathways, network pharmacology was employed to further pinpoint key target genes, and molecular docking was used to validate their binding affinity to quercetin, achieving a multi-layered elucidation of its therapeutic action [21].

There is a bidirectional relationship between lipid metabolism and the gut microbiota. The gut microbiota profoundly regulates lipid metabolism through the “gut–liver axis”. Some specific taxa such as *Akkermansia muciniphila* enhance the intestinal barrier integrity and glucagon-like peptide-1 (GLP-1) secretion to improve insulin sensitivity [22], whereas an elevated Firmicutes/Bacteroidetes ratio exacerbates lipid accumulation through fermentation of excessive short-chain fatty acids (SCFAs) [23]. Dietary polyphenols can remodel this interaction via “microbiota–host” co-metabolism. For instance, tea polyphenols alleviate high-fat-diet-induced metabolic endotoxemia by enriching *Akkermansia muciniphila* and upregulating intestinal tight junction protein expression [24]. Similarly, blueberry procyanidins promote the growth of *Bacteroides* spp., enhance SCFA production, and suppress hepatic de novo lipogenesis via activation of the AMPK pathway [25,26]. Notably, procyanidins can be metabolized by specific gut microbes (such as *Clostridium saccharogumia*) into phenylpropionic acid derivatives. These metabolites not only directly inhibit hepatic *FASN* expression but also restructure the microbial community, establishing a positive feedback loop [27]. While multi-omics studies have preliminarily revealed the regulatory networks of procyanidins in obesity-related glucose–lipid metabolism and atherosclerosis, previous research has predominantly relied on single- or dual-omics analyses such as metabolomics–network pharmacology or metabolomics–gut microbiota [14,15,28,29]. There has been no research that integrates metabolomics, network pharmacology, and gut microbiota analysis to dissect the mechanism by which procyanidins, particularly PSPs, synergistically resolve lipid dysregulation.

Therefore, this study employed a high-fat-diet-induced obesity mouse model and combined metabolomics, network pharmacology, and microbiota profiling analysis to decipher the lipid regulatory mechanisms of PSP. Specifically, the study aims to (1) reveal the metabolic pathways modulated by PSP in lipid metabolism disorders by hepatic metabolomics; (2) construct a “PSP–target–obesity” interaction network to identify the core regulatory genes and further validate them by qPCR; and (3) analyze the impact of PSP on obesity-associated gut microbiota composition and function. The findings are expected to provide a theoretical basis for the clinical application of PSP and development of strategies for repurposing agricultural waste into therapeutic agents for metabolic diseases.

## 2. Materials and Methods

### 2.1. Materials

#### 2.1.1. Chemicals and Reagents

Procyanidins from peanut skin (PSPs, oligomeric procyanidins ≥ 95%) were provided by Xi’an Fenghe Biotechnology Co., Ltd. (Xi’an, China). Orlistat (purity ≥ 98%, CAS 96829-58-2) and sodium carboxymethyl cellulose (CMC-Na, food-grade, CAS 9004-32-4) were purchased from Shanghai Yuanye Bio-Technology Co., Ltd. (Shanghai, China) and Sinopharm Chemical Reagent Co., Ltd. (Shanghai, China), respectively. Lipid profile assay kits (TC-A111, TG-A110, HDL/LDL-A112/A113) and liver function kits (ALT-C009, AST-C010) were obtained from Nanjing Jiancheng Bioengineering Institute (Nanjing, China). Ammonium formate (≥99.0%) and methyl tert-butyl ether (HPLC-grade) were sourced from Sigma-Aldrich (Shanghai, China) and Tedia (Shanghai, China), respectively.

#### 2.1.2. Instruments and Equipment

The following instruments were used: constant-temperature water bath (HH-4, Guohua Electric Co., Ltd., Wuhan, China), rotary evaporator (RE52-99, Shanghai Yarong Biochemical Instrument Factory, Shanghai, China), circulating water vacuum pump (SHB-IV, Guohua Electric Co., Ltd., Wuhan, China), magnetic stirrer (DF-101S, Wuhan Cole Instrument Co., Ltd., Wuhan, China), autoclave (YXQ-LS-50G, Shanghai Boxun Industrial Co., Ltd., Shanghai, China), full-wavelength microplate reader (Multiskan GO, Thermo Fisher Scientific, Shanghai, China), analytical balance (AL204, Mettler-Toledo Instruments, Guangzhou, China), Hypersil GOLD C18 column (100 × 2.1 mm, 5 µm; Thermo Fisher Scientific, Shanghai, China), UPLC system (UltiMate 3000, Thermo Fisher Scientific, Shanghai, China), high-speed centrifuge (CF1524R, Sartorius, Beijing, China), inverted microscope (Eclipse TS100, Nikon, Shanghai, China), and LC-MS system (AB SCIEX Triple TOF 5600, Shanghai, China; ACQUITY UHPLC, Waters, Shanghai, China).

### 2.2. Animal Experimental Design

**Figure 1:**
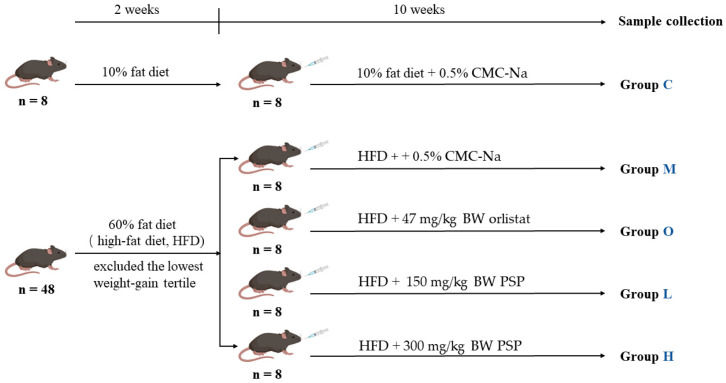
Schematic diagram of animal experimental.

Fifty-six SPF-grade male C57BL/6J mice (6 weeks old, 18–20 g) were purchased from Henan Suke Beisi Biotechnology Co., Ltd. (SYXK 2020-0084, Zhengzhou, China). Mice were maintained under controlled environmental conditions (20 ± 2°C, 40–60%, 12 h light/dark cycle) in individually ventilated polycarbonate cages (290 × 178 × 160 mm; four mice/cage). Bedding consisted of high-temperature-sterilized aspen wood shavings (~2 cm depth), replaced every 48 h. Throughout the study, mice had *ad libitum* access to diet and water. As shown in Figure 1, mice were divided into a low-fat control group (Group C, *n* = 8) fed with a 10% fat diet (TP23302, Nantong Trophic, Nantong, China) and a high-fat diet (HFD) group (*n* = 48) fed with a 60% fat diet (TP23300, Nantong Trophic). After two weeks of HFD feeding, the lowest weight-gain tertile was excluded. The remaining 32 mice were randomly divided into four groups (*n* = 8/group), including the HFD model (Group M), high-dose PSP (Group H, 300 mg/kg BW), low-dose PSP (Group L, 150 mg/kg BW), and orlistat (Group O, 47 mg/kg BW). All test substances were suspended in 0.5% (*w*/*v*) CMC-Na solution and administered daily via oral gavage for 10 weeks [14,15,30,31]. The body weight and energy intake were recorded weekly. All procedures were approved by the Laboratory Animal Centre of Huazhong Agricultural University (HZAUMO-2024-0087).

### 2.3. Biochemical Analysis

Following retro-orbital blood collection, blood samples were centrifuged at 3000× *g* for 10 min at 4 °C to collect the serum. The serum levels of TC, TG, HDL-C, LDL-C, ALT, and AST were measured using commercial assay kits according to the manufacturer’s protocols.

For histological analysis, liver tissues fixed in 4% paraformaldehyde were dehydrated in graded ethanol, embedded in paraffin, and sectioned into 4 µm thick slices. Sections were deparaffinized, rehydrated, and stained with hematoxylin and eosin (H&E) for morphological evaluation. Additionally, fixed liver tissues were embedded in optimal cutting temperature compound, rapidly frozen, sectioned at an 8 µm thickness, and stained with Oil Red O after isopropanol incubation. Finally, observation and photography were performed under a 200× optical microscope.

### 2.4. Hepatic Metabolomics Analysis

#### 2.4.1. Sample Preparation

Following the description of Wen [32], the liver tissue (40 mg) was homogenized in 800 µL of ultrapure water. Methanol (600 µL) and methyl tert-butyl ether (2000 µL) were added, vortexed for 1 min, and centrifuged (2400× *g*, 15 min). The upper phase was lyophilized and reconstituted in 200 µL of methanol/dichloromethane (50:50, *v*/*v*) containing 5 mM ammonium acetate for lipidomics. The lower phase was stored at −20 °C overnight and centrifuged (3000× *g*, 10 min), and the supernatant was collected for metabolomics. Pooled quality control (QC) samples (10 µL aliquots per individual sample) and solvent-processed blanks accompanied each analysis batch.

#### 2.4.2. Lipidomics

Following the description of Wen [32], chromatographic separation was performed using a Hypersil GOLD C18 column with mobile phases: (A) 0.1% formic acid and 5 mM ammonium formate in acetonitrile/water (3:2, *v*/*v*); and (B) 0.1% formic acid and 5 mM ammonium formate in isopropanol/acetonitrile (9:1, *v*/*v*). The gradient elution was 80% A (0–0.5 min), 60% A (1 min), 40% A (3 min), 2% A (16 min), and 80% A (17–20 min). The flow rate was 0.3 mL/min, and the injection volume was 5 µL. For both lipidomics and metabolomics, the conditions were set as follows. The column temperature was maintained at 40 °C; the autosampler temperature was set at 5 °C; samples were analyzed in a randomized injection order within each experimental group, with a single injection per sample; one QC sample and one blank sample were injected after every eight experimental sample injections; and mass spectrometry was performed using electrospray ionization (ESI) in both positive and negative ion modes, with an *m*/*z* scan range of 100–1500.

#### 2.4.3. Metabolomics

Following the conditions described by Wen [32], the positive ion mode was mobile phase A (0.1% formic acid in water) and B (acetonitrile). The negative ion mode was A (5 mM ammonium acetate in water) and B (acetonitrile). The gradient was 95% A (0–1.5 min), 75% A (2.5 min), 50% A (5 min), 30% A (8 min), 20% A (10 min), 5% A (13–17 min), and 95% A (18–20 min). The flow rate was 0.3 mL/min, and the injection volume was 2 µL.

#### 2.4.4. Data Processing

Raw UPLC-Q-TOF-MS data were processed using Progenesis QI (Waters, Milford, MA, USA) for peak alignment, deconvolution, and log transformation. Metabolite identification required meeting the following criteria: exact mass error < 5 ppm, MS/MS spectral matches to HMDB (https://hmdb.ca/) and LIPID MAPS (https://lipidmaps.org/) databases, and intensity CV < 30%. Multivariate analysis and pathway mapping were performed on MetaboAnalyst 5.0 (https://www.metaboanalyst.ca/). Differential metabolites were screened via oPLS-DA with VIP >1, ANOVA *p* < 0.05, and fold change (FC) >1.5 or <0.67.

### 2.5. Network Pharmacology Analysis

The potential bioactive components of PSP were identified by Zhao et al. [33] and Chen [30]. Targets were predicted using TCMSP (https://www.tcmsp-e.com/), SwissTargetPrediction (http://swisstargetprediction.ch/), and PharmMapper (https://lilab-ecust.cn/pharmmapper/, accessed on 27 May 2025). Disease targets (“obesity”, “overweight”) were retrieved from GeneCards (https://www.genecards.org/), DisGeNET (https://disgenet.com/), and OMIM (https://www.omim.org/). Venn (https://jvenn.toulouse.inra.fr/app/example.html, accessed on 27 May 2025) analysis was used to identify the shared targets, and STRING/Cytoscape 3.6.0 was used to construct the interaction networks. KEGG and GO enrichment analyses were performed via DAVID (https://davidbioinformatics.nih.gov/tools.jsp, accessed on 27 May 2025).

### 2.6. Integrated Analysis of Metabolomics and Network Pharmacology 

Following the conditions described by Sun et al. [21], a compound–reaction–enzyme–gene (CREG) network was built using MetScape (Cytoscape 3.6.0). The overlapping targets from network pharmacology and metabolomics were validated by qPCR.

### 2.7. Quantitative Real-Time PCR

RNA-specific primers were designed using PrimerBank (https://pga.mgh.harvard.edu/primerbank/, accessed on 27 May 2025) with the following parameters: melting temperature (Tm) = 60 ± 3 °C, GC content = 40–60%, amplicon length = 80–150 bp, and primer length = 15–25 nt. All primer pairs were designed to span exon–exon junctions to eliminate genomic DNA amplification. The primers were rigorously validated against the reference genome using the Basic Local Alignment Search Tool (BLAST) to minimize off-target amplification risks. Validated primer sequences (Table A2) were chemically synthesized and purified via polyacrylamide gel electrophoresis (PAGE) by Tsingke Biotechnology Co., Ltd. (Beijing, China). RNA extraction, reverse transcription, and qPCR were performed using AFTSpin, ABScript III, and SYBR Green kits, respectively.

### 2.8. Gut Microbiota Analysis

The cecum contents were aseptically collected from mice, immediately frozen in liquid nitrogen, and stored at −80 °C. Following the extraction of genomic DNA from the cecal contents, the DNA was analyzed by agarose gel electrophoresis. PCR amplification was performed using primers 338F (5′-ACTCCTACGGGAGGCAGCAG-3′) and 806R (5′-GGACTACHVGGGTWTCTAAT-3′), along with TransGen AP221-02 reagents, TransStart Fastpfu DNA Polymerase, and an ABI GeneAmp^®^ 9700 PCR system (Shanghai, China). The PCR products were subsequently analyzed by agarose gel electrophoresis. Target DNA fragments were excised and purified using an AxyPrep DNA Gel Extraction Kit (Axygen Biosciences, Shanghai, China), followed by elution with Tris-HCl buffer. The purified products were re-examined via 2% agarose gel electrophoresis. Based on preliminary electrophoretic quantification, PCR products were quantified using the QuantiFluor™-ST Blue Fluorescence Quantification System (Promega, Beijing, China). Subsequently, the products were pooled in appropriate ratios according to sequencing requirements. Libraries were constructed using a TruSeq™ DNA Sample Prep Kit (Shanghai, China) and subjected to sequencing. Paired-end (PE) reads obtained from sequencing were first assembled based on the overlapping regions, followed by quality control, filtering, and demultiplexing by sample. Operational taxonomic unit (OTU) clustering and taxonomic analyses were performed. All experimental procedures, including DNA extraction and sequencing, were conducted by Majorbio Bio-pharm Technology Co., Ltd. (Shanghai, China). For further statistical and visualization analyses, the obtained data were processed using the Majorbio Cloud Platform (https://cloud.majorbio.com).

### 2.9. Statistical Analysis

Data were expressed as mean ± SEM. ANOVA and Tukey’s test (GraphPad Prism 9.0) were used for group comparisons (*p* < 0.05). Network enrichment employed hypergeometric testing with FDR correction.

## 3. Results

### 3.1. Effects of PSP on Body Weight, Lipid Profile, and Liver Function

As shown in Table 1, mice in Group M exhibited rapid weight gain, whereas PSP and orlistat intervention significantly attenuated the HFD-induced weight gain (*p* < 0.05). After 12 weeks, Group O mice showed significantly lower body weight than Group M mice (*p* < 0.05) while showing a comparable body weight to Group C, despite their higher energy intake than Group M and C (*p* < 0.05). Both Group L and H displayed significantly lower body weight than Group M (*p* < 0.05), and their energy intake was similar to that of Group M but higher than that of Group C (*p* < 0.05).

**Figure 2:**
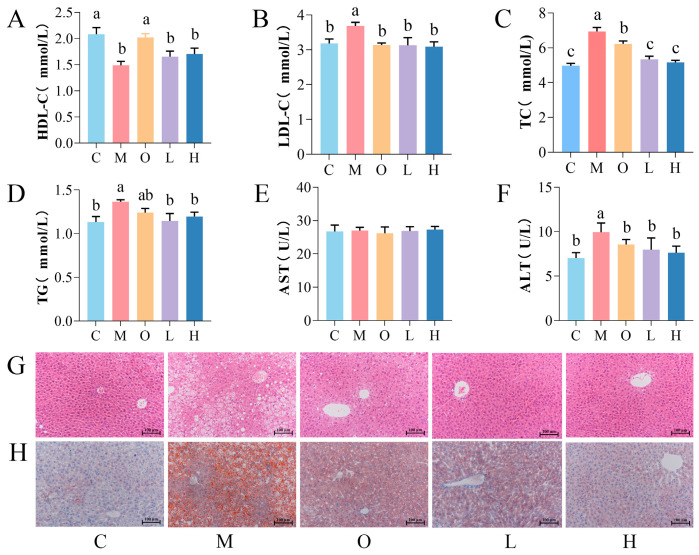
Effects of PSP intervention on the body weight, blood lipids, and hepatic function in HFD mice (*n* = 8). HDL-C (**A**), LDL-C (**B**), TC (**C**), TG (**D**), AST (**E**), ALT (**F**), H&E (**G**), and Oil Red O (**H**) staining of mice liver (200×). Groups: C (10% fat diet), M (60% HFD), L (HFD + 150 mg/kg PSP), H (HFD + 300 mg/kg PSP), O (HFD + 47 mg/kg orlistat). Different letters indicate significant differences between groups (*p* < 0.05).

Serum analysis revealed that Group M had significantly lower HDL-C and higher LDL-C, TG, and TC levels compared with Group C (*p* < 0.05; Figure 2A–D). PSP intervention (Group L and H) restored the LDL-C, TG, and TC to normal levels comparable to those of Group C (*p* > 0.05; Figure 2A–D), indicating the efficacy of PSP in ameliorating HFD-induced dyslipidemia.

While AST activity showed no significant differences among groups (Figure 2E), Group M showed higher ALT activity than Group C (*p* < 0.05), which was reversed by PSP intervention (Group L and H; Figure 2F). Histopathological analysis via H&E staining revealed a phenomenon of vacuolation and loose organization of hepatic architecture in Group M, whereas PSP and orlistat intervention restored the tissue integrity (Figure 2G). Oil Red O staining further confirmed reduced lipid droplet accumulation in the PSP-treated groups (Figure 2H), demonstrating the protective effects of PSP against HFD-induced hepatic steatosis.

### 3.2. Hepatic Metabolomics Analysis

**Figure 3:**
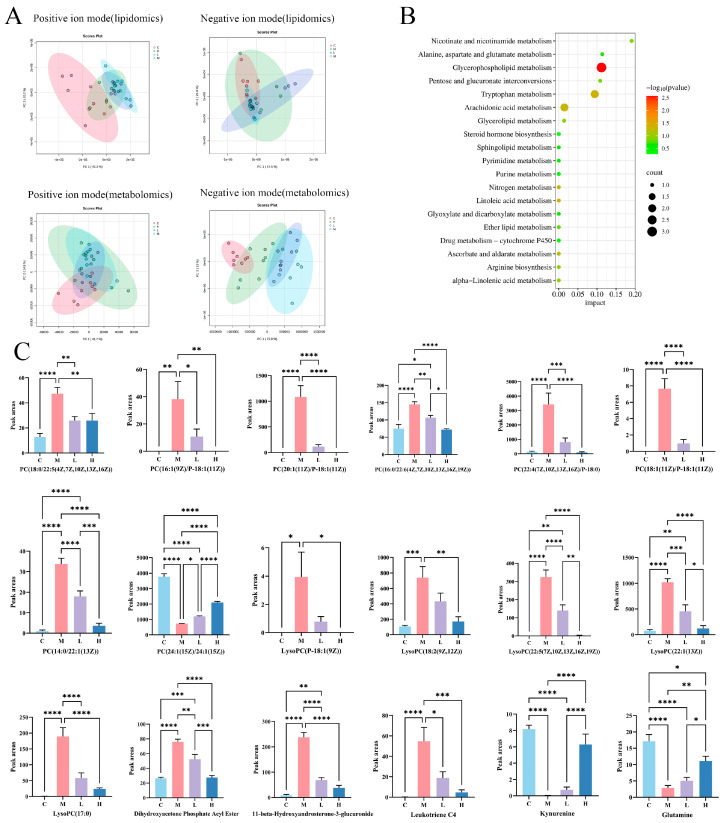
Multivariate statistical analysis of hepatic lipidomics and metabolomics in mice (*n* = 8). PCA score plot (**A**), metabolomics pathway analysis (**B**), and peak areas of metabolites (**C**). Groups: C (10% fat diet), M (60% HFD), L (HFD + 150 mg/kg PSP), H (HFD + 300 mg/kg PSP). “*”, “**”, “***”, and “****” represent significance at the levels of *p* < 0.05, *p* < 0.01, *p* < 0.001, and *p* < 0.0001, respectively.

PCA showed a clear separation between Group C and M, reflecting that HFD induced metabolic perturbations in the mice (Figure 3A). PSP intervention partially reversed these perturbations. A total of 61 differential metabolites were identified (Table A3). Pathway analysis highlighted five key pathways, including glycerophospholipid, linoleic acid, tryptophan, nitrogen, and arachidonic acid metabolism (Figure 3B). The associated metabolites included phosphatidylcholine, 1-acyl-*sn*-glycero-3-phosphocholine, dihydroxyacetone phosphate acyl ester, leukotriene C4, kynurenine, and glutamine. In contrast to its effects on PC(24:1(15Z)/24:1(15Z)), kynurenine, and glutamine, PSP intervention significantly attenuated the HFD-induced increases in other metabolites (Figure 3C).

### 3.3. Network Pharmacology Analysis

**Figure 4:**
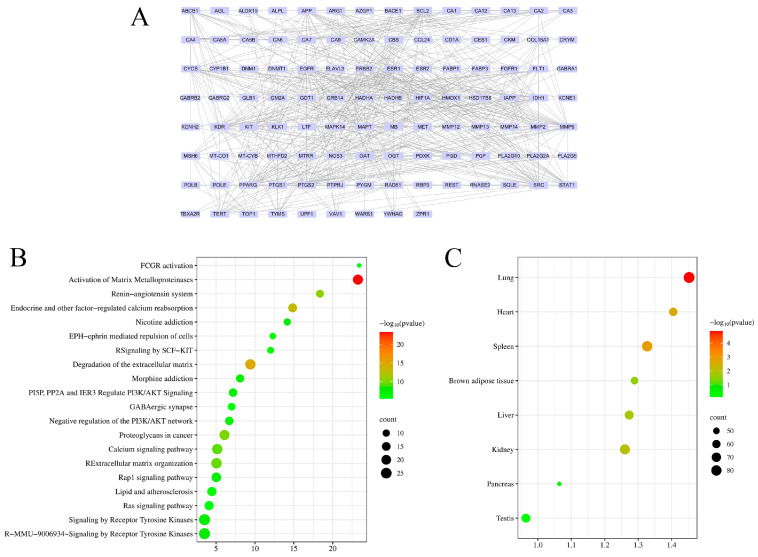
Network pharmacological analysis of PSP and obesity. Network of shared target genes (**A**), KEGG (**B**), and tissue expression enrichment (**C**).

An integrative analysis of PSP-derived bioactive compounds and obesity-related targets identified 114 key genes (Table A4). STRING and Cytoscape analyses revealed a gene interaction network (Figure 4A), with KEGG enrichment highlighting the activation of matrix metalloproteinases (Figure 4B). Tissue-specific expression analysis indicated that these genes were predominantly related to liver and brown adipose tissue (Figure 4C).

### 3.4. Integrated Analysis of Metabolomics– and Network Pharmacology

**Figure 5:**
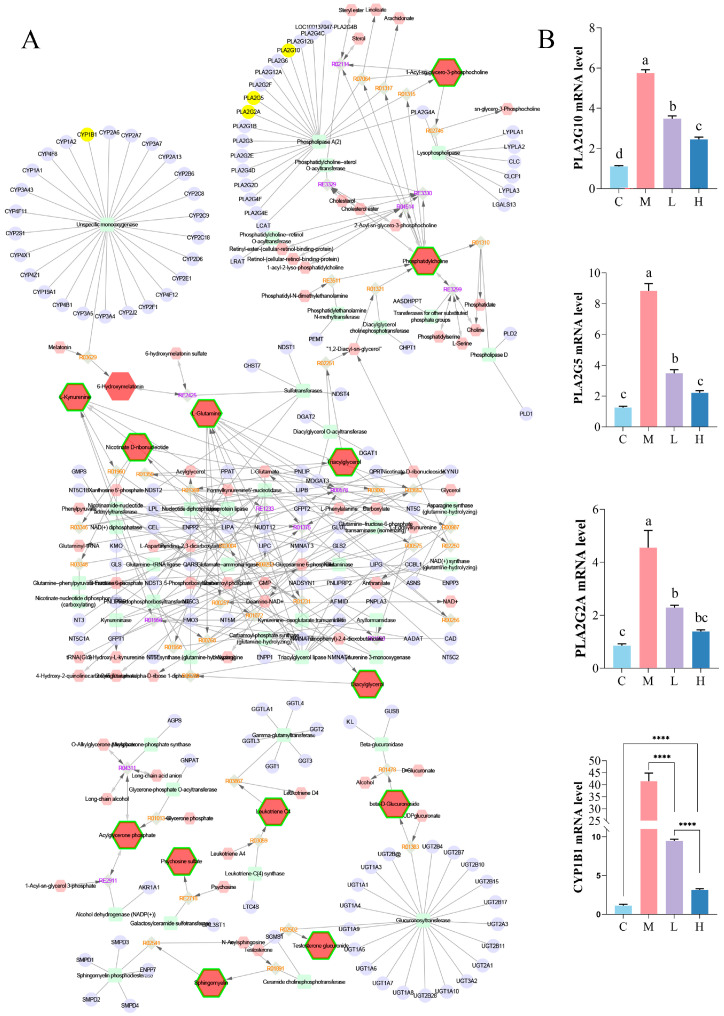
Integrated Analysis of Metabolomics– and Network Pharmacology (*n* = 8). CREG network (**A**), mRNA Levels of *Pla2g1*, *Pla2g5*, *Pla2g2a*, and *Cyp1b1* (**B**) in the liver tissue. Red hexagons, gray diamonds, green rectangles, purple circles, and yellow circles represent active compounds, reactions, enzymes, genes, and target genes, respectively. Groups: C (10% fat diet), M (60% HFD), L (HFD + 150 mg/kg PSP), H (HFD + 300 mg/kg PSP). Different letters indicate significant differences between groups (*p* < 0.05). “****” indicates *t* test *p* < 0.0001 is significant.

A compound–reaction–enzyme–gene (CREG) network (Figure 5A) was constructed by integrating the 61 differential metabolites and network pharmacology-predicted targets. Intersection analysis identified *Pla2g10*, *Pla2g5*, *Pla2g2a*, and *Cyp1b1* as the four core targets. qPCR confirmed that PSP suppressed the HFD-induced upregulation of these genes (Figure 5B), which validated the results of the integrated metabolomics and network pharmacology analyses.

### 3.5. Effect of PSP on Gut Microbiota Composition

**Figure 6:**
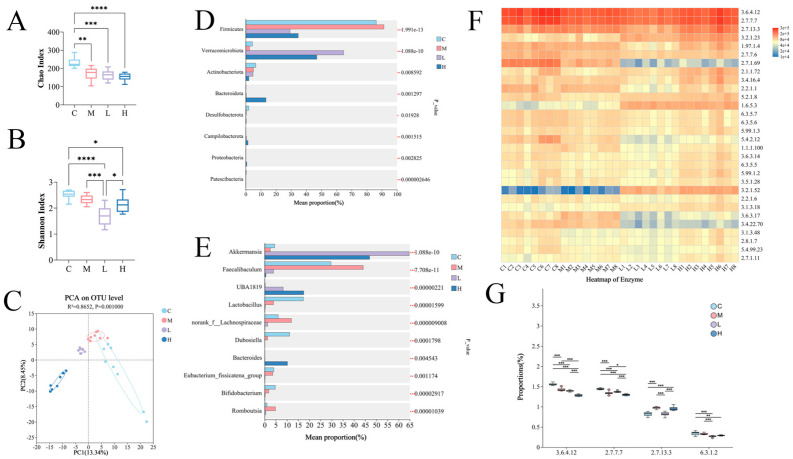
Effects of PSP intervention on intestinal microbiota in cecum contents (*n* = 8). Chao (**A**), Shannon (**B**), PCA (**C**), differences in the relative abundance of intestinal microbiota at the phylum level (**D**) and genus level (**E**) between different groups, enzyme heatmap (**F**), proportion analysis of EC 3.6.4.12, EC 2.7.7.7, EC 2.7.13.3, and EC 6.3.1.2 (**G**). Groups: C (10% fat diet), M (60% HFD), L (HFD + 150 mg/kg PSP), H (HFD + 300 mg/kg PSP). “*”, “**”, “***”, and “****” represent significance at the levels of *p* < 0.05, *p* < 0.01, *p* < 0.001, and *p* < 0.0001, respectively.

HFD significantly decreased the Chao and Shannon indices (*p* < 0.05 vs. Group C; Figure 6A,B), indicating that it diminishes microbial richness and diversity. PSP failed to restore α-diversity but altered β-diversity (Figure 6C). In addition, PSP intervention significantly altered gut microbiota composition by increasing the relative abundance of *Akkermansia* within Verrucomicrobia while reducing that of *Faecalibaculum*, *norank_f__Lachnospiraceae,* and *Romboutsia* within Firmicutes. High-dose PSP resulted in a significant enrichment of *Bacteroides* within Bacteroidota (Figure 6D,E). Functional prediction via PICRUSt2 revealed that the enzyme activity profile was predominated by DNA helicase (EC 3.6.4.12), DNA polymerase (EC 2.7.7.7), and histidine kinase (EC 2.7.13.3) (Figure 6F). Metabolomics analysis revealed that glutamine synthetase (EC 6.3.1.2) was a pivotal enzyme driving the metabolic divergence between Group M and the PSP-treated groups, and thus, it is necessary to further investigate the effect of PSP on this enzyme (Figure 6F). PSP altered the proportions of these enzymes (*p* < 0.05) (Figure 6G).

## 4. Discussion

Although previous studies have revealed the regulatory networks of procyanidins on lipid metabolism through multi-omics approaches, there has been a lack of in-depth research on PSP. Previous studies have been predominantly focused on single or dual omics analyses (such as metabolomics–network pharmacology, metabolomics–gut microbiota) [14,15,28,29], failing to systematically elucidate the integrative mechanism by which procyanidins, particularly PSP, ameliorate lipid dysregulation by integrating metabolomics, network pharmacology, and gut microbiota analysis. In this study, we integrated these approaches to systematically uncover the molecular network through which PSP alleviates lipid metabolism disorders. The results not only validated its therapeutic potential but also provided novel insights into the synergistic regulation of gut microbiota and metabolic pathways.

Metabolomic analysis revealed that PSP significantly modulates hepatic lipid dynamics via affecting the glycerophospholipid, linoleic acid, tryptophan, nitrogen, and arachidonic acid metabolism. Pharmacology screening indicated that PSP targets *PLA2G10*, *PLA2G5*, *PLA2G2A*, and *CYP1B1* to regulate glycerophospholipid, linoleic acid, arachidonic acid, and tryptophan metabolism. In contrast, grape seed procyanidins primarily target *PLA2G4A* [34], while hawthorn procyanidins act via the AMPK pathway [35]. The phospholipase A2 (PLA2) family, which comprises critical enzymes in phospholipid hydrolysis, converts phospholipids into absorbable free fatty acids, and their activity is positively correlated with the serum level of free fatty acids [36,37]. Here, PSP intervention was found to significantly reduce the levels of phosphatidylcholine and 1-acyl-sn-glycero-3-phosphocholine, indicating that PSP lowers systemic lipid content, probably by inhibiting lipid absorption and then downregulating *PLA2G10*, *PLA2G5*, and *PLA2G2A* expression, ultimately modulating glycerophospholipid, linoleic acid, and arachidonic acid metabolism. CYP1B1 exacerbates hepatic lipid accumulation by inducing lipid phagocytosis [38], while enhanced aryl hydrocarbon receptor (AhR) activation suppresses *CYP1B1* expression [39]. Tryptophan degradation metabolites (such as kynurenine) can activate AhR [40,41], indicating that PSP inhibits *CYP1B1* expression by elevating the kynurenine level, thereby mitigating hepatic lipid accumulation.

Similar to tea polyphenols and grape seed procyanidin extracts [32,42], PSP also failed to reverse the HFD-induced decline in microbial α-diversity. However, it could selectively remodel the gut microbiota by enriching beneficial bacteria such as *Akkermansia* and *Bacteroides* while suppressing harmful bacteria within Firmicutes. Previous studies have demonstrated that *Akkermansia* enhances energy expenditure and reduces visceral adiposity by secreting the P9 protein to activate brown adipose tissue (BAT) thermogenesis [43]. *Bacteroides* enhances host metabolism via polysaccharide digestion and SCFA production [44,45]. *Bacteroides uniformis* converts host primary bile acids into the secondary bile acid 3-succinylated cholic acid, which promotes the growth of *Akkermansia muciniphila* and subsequently modulates lipid metabolism [46]. Conversely, a reduction in Firmicutes abundance may attenuate excessive SCFA fermentation and lipid synthesis [47,48]. Notably, PSP selectively altered β-diversity rather than restoring α-diversity [42,49], highlighting its targeted modulatory effects.

Hepatic metabolomic analysis revealed that PSP intervention increased the glutamine level. Glutamine participates in α-ketoglutarate synthesis, thereby enhancing mitochondrial TCA cycle efficiency and promoting fatty acid oxidation [50,51,52,53]. PICRUSt-based functional prediction of the gut microbiota further indicated that PSP may modulate glutamine-associated nitrogen and lipid metabolism by downregulating gut microbial glutamine synthetase activity. Similar to the mechanism of inulin fiber, which reverses microbiota-derived free bile acid agonism via the BA-MCY axis to promote bile acid synthesis gene expression and regulate hepatobiliary metabolism in vivo [54], PSP appears to exert its effects through gut-microbiota-mediated glutamine regulation. When gut microbiota perturbation reduces intestinal glutamine synthetase activity, the liver compensatorily elevates the hepatic glutamine level [50,55,56,57]. These findings suggest that PSP may indirectly enhance hepatic glutamine by reducing its intestinal availability, thereby attenuating lipid accumulation and stimulating energy expenditure, which offers novel insights into the gut–liver axis in lipid metabolic regulation.

Given the established role of dysregulated lipid metabolism in pathologies such as non-alcoholic fatty liver disease (NAFLD) and atherosclerosis [58,59], the therapeutic potential of PSP extends beyond basic lipid reduction. Its modulation of lipid homeostasis may deliver positive therapeutic effects for these conditions. NAFLD—a chronic liver disorder characterized by hepatocellular lipid accumulation [60]—can be ameliorated by PSP through suppression of CYP1B1-mediated lipid phagocytosis and enhanced hepatic α-ketoglutarate synthesis via glutamine metabolism, indicating its utility in NAFLD management. Xu et al. confirmed PSP’s capacity to reduce atherosclerotic plaques [15]. Integrated with our data, this benefit likely originates from PSP’s systemic lipid-lowering effects, which inhibit lipid-deposition-driven plaque formation, thereby attenuating atherosclerosis. Since cancer progression is profoundly modulated by lipid metabolism [61], PSP may influence oncogenesis through lipid metabolic regulation. Notably, PICRUSt-based functional analysis revealed that PSP intervention suppresses DNA-synthesis-related enzymatic activity, potentially underpinning its anti-carcinogenic mechanisms.

## 5. Conclusions

This study systematically deciphered the molecular mechanisms by which PSP ameliorates lipid metabolism disorders by integrating multi-omics approaches. Comprehensive analysis revealed that PSP reduces hepatic lipid content, elevates the hepatic kynurenine level, and downregulates the expression of *PLA2G10*, *PLA2G5*, *PLA2G2A*, and *CYP1B1*, thereby modulating lipid-metabolism-related pathways, including glycerophospholipid, linoleic acid, arachidonic acid, and tryptophan metabolism. PSP primarily influences lipid metabolism by restructuring the composition of *Akkermansia*, *Bacteroidota*, and Firmicutes while concurrently affecting glutamine synthetase activity. In summary, PSP downregulates *PLA2G10*, *PLA2G5*, *PLA2G2A*, and *CYP1B1* expression, enhances the hepatic glutamine level via gut-microbiota-mediated modulation, and enriches SCFA-producing and energy-expenditure-stimulating bacterial communities, which collectively regulate lipid absorption, accumulation, and catabolism. This study provides a theoretical basis for the regulation of lipid metabolism by PSP, establishing a “metabolome–target–microbiota” research paradigm. It not only offers conceptual guidance and theoretical support for research on PSP’s improvement of lipid-metabolism-related diseases but also provides methodological approaches for studying bioactive compounds such as procyanidins.

## Figures and Tables

**Table 1:** Effects of PSP on the body weight, energy intake, and water intake of mice.

Group	C	M	O	L	H
Initial body weight/g	21.51 ± 0.69 ^b^	24.36 ± 1.37 ^a^	23.94 ± 0.77 ^a^	24.03 ± 0.45 ^a^	24.21 ± 0.19 ^a^
Final body weight/g	25.94 ± 1.30 ^b^	32.94 ± 5.40 ^a^	28.74 ± 1.24 ^b^	28.08 ± 1.70 ^b^	27.53 ± 0.67 ^b^
Body weight gain/g	4.43 ± 0.71 ^b^	8.57 ± 4.19 ^a^	4.80 ± 0.89 ^b^	4.05 ± 1.68 ^b^	3.31 ± 0.72 ^b^
Energy intake (kcal/d/g)	9.37 ± 0.50 ^c^	11.10 ± 0.08 ^b^	12.63 ± 1.01 ^a^	10.53 ± 0.10 ^b^	10.91 ± 0.92 ^b^

Note: Different letters in the same line indicate significant differences between groups (*p* < 0.05, *n* = 8). Groups: C (10% fat diet), M (60% HFD), L (HFD + 150 mg/kg PSP), H (HFD + 300 mg/kg PSP), O (HFD + 47 mg/kg orlistat).

## Data Availability

All other data that support the findings of this study are available from the first author upon reasonable request.

## Abbreviations

The following abbreviations are used in this manuscript:

AhR	Aryl hydrocarbon receptor
AMPK	Adenosine-monophosphate-activated protein kinase
BAT	Brown adipose tissue
CREG	Chemical–reaction–enzyme–gene
GLP-1	Glucagon-like peptide-1
HFD	High-fat diet
PPARγ	Peroxisome proliferator-activated receptor gamma
PSP	Peanut-skin-derived procyanidins
SCFA	Short-chain fatty acids

## Appendix A

**Table A1:** Composition of experimental diets for animal study.

Feed Code	TP23300	TP23302
Protein (casein, L-cystine)/g	276	194
Carbohydrate (dextrin, sucrose)/g	250	
Carbohydrate (dextrin, sucrose, corn starch)/g		673
Fat (soybean oil, lard)/g	341	40
Fiber (cellulose)/g	68	48
Mineral and vitamin mixture/g	65	45
Antioxidant (TBHQ)/g	0.07	0.01
Total/g	1000	1000

**Table A2:** Primer sequence.

Gene Name	Forward Primer (5′-3′)	Reverse Primer (5′-3′)
*Pla2g10*	GTGCAGGTGTGACGAGGAG	CACTTGGGAGAGTCCTTCTCA
*Pla2g5*	CCAGGGGGCTTGCTAGAAC	AGCACCAATCAGTGCCATCC
*Pla2g2a*	TGGCTCAATACAGGACCAAGG	GTGGCATCCATAGAAGGCATAG
*Cyp1b1*	CACCAGCCTTAGTGCAGACAG	GAGGACCACGGTTTCCGTTG

**Table A3:** C vs. M and PSP vs. M share analyzable differential metabolites.

Number	HMDB	KEGG ID	Metabolites	Retention Time (s)
1	HMDB0000305	C17276	Vitamin A	4.40_269.2272*m*/*z*
2	HMDB0000445	C06768	Alpha-tetrasaccharide	8.59_672.2381*m*/*z*
3	HMDB0000641	C00064	Glutamine	1.05_181.0387*m*/*z*
4	HMDB0000684	C00328	Kynurenine	1.34_231.0733*m*/*z*
5	HMDB0000933	C16308	Traumatic acid	1.95_246.1700*m*/*z*
6	HMDB0001045	C16041	Enkephalin L	9.39_578.2563*m*/*z*
7	HMDB0001132	C01185	Nicotinic acid mononucleotide	0.89_731.1126*m*/*z*
8	HMDB0001140	C01545	Octanal	3.09_111.1175*m*/*z*
9	HMDB0001198	C02166	Leukotriene C4	8.76_606.2831*m*/*z*
10	HMDB0002937	C10188	Tamarixetin	1.29_317.0681*m*/*z*
11	HMDB0003193	C11134	Testosterone glucuronide	5.73_499.2103*m*/*z*
12	HMDB0004159	C05793	L-Urobilin	4.12_617.3291*m*/*z*
13	HMDB0005398	C00422	TG(18:0/18:0/20:4(5Z,8Z,11Z,14Z))	18.72_893.7915*m*/*z*
14	HMDB0006061	C05595	4-Hydroxyphenylacetylglutamic acid	1.07_262.0725*m*/*z*
15	HMDB0006575	C06372	Lacto-N-biose I	18.61_366.1412*m*/*z*
16	HMDB0007112	C00165	DG(16:0/20:4(5Z,8Z,11Z,14Z)/0:0)	18.57_616.5089n
17	HMDB0007224	C00165	DG(18:1(9Z)/20:1(11Z)/0:0)	12.44_683.5355*m*/*z*
18	HMDB0007228	C00165	DG(18:1(9Z)/20:4(5Z,8Z,11Z,14Z)/0:0)	10.49_625.5188*m*/*z*
19	HMDB0007887	C00157	PC(14:0/22:1(13Z))	8.03_826.5726*m*/*z*
20	HMDB0007991	C00157	PC(16:0/22:6(4Z,7Z,10Z,13Z,16Z,19Z))	8.25_806.5698*m*/*z*
21	HMDB0008029	C00157	PC(16:1(9Z)/P-18:1(11Z))	9.76_722.5494*m*/*z*
22	HMDB0008055	C00157	PC(18:0/22:5(4Z,7Z,10Z,13Z,16Z))	7.09_818.6075*m*/*z*
23	HMDB0008095	C00157	PC(18:1(11Z)/P-18:1(11Z))	4.34_787.6343*m*/*z*
24	HMDB0008326	C00157	PC(20:1(11Z)/P-18:1(11Z))	11.53_778.6136*m*/*z*
25	HMDB0008653	C00157	PC(22:4(7Z,10Z,13Z,16Z)/P-18:0)	10.84_802.6133*m*/*z*
26	HMDB0008816	C00157	PC(24:1(15Z)/24:1(15Z))	4.35_992.7452*m*/*z*
27	HMDB0010328	C03033	Tyramine glucuronide	8.67_685.2440*m*/*z*
28	HMDB0010347	C03033	Octanoylglucuronide	18.97_357.0953*m*/*z*
29	HMDB0010351	C05643	11-beta-Hydroxyandrosterone-3-glucuronide	8.86_481.2455*m*/*z*
30	HMDB0010386	C04230	LysoPC(18:2(9Z,12Z))	8.76_556.2811*m*/*z*
31	HMDB0010399	C04230	LysoPC(22:1(13Z))	10.86_577.4089n
32	HMDB0010403	C04230	LysoPC(22:5(7Z,10Z,13Z,16Z,19Z))	9.81_568.3387*m*/*z*
33	HMDB0010408	C04230	LysoPC(P-18:1(9Z))	9.08_542.3018*m*/*z*
34	HMDB0011750	C03372	Dihydroxyacetone phosphate acyl ester	0.92_178.9747*m*/*z*
35	HMDB0012107	C00550	SM (d18:1/24:1(15Z))	18.61_813.6849*m*/*z*
36	HMDB0012108	C04230	LysoPC (17:0)	8.90_509.3504n
37	HMDB0013046	C02744	Psychosine sulfate	6.86_540.2838*m*/*z*
38	HMDB0014363	C07663	Moxifloxacin	7.86_436.1453*m*/*z*
39	HMDB0014383	C07263	Nevirapine	1.24_303.0645*m*/*z*
40	HMDB0014498	C07777	Buclizine	5.34_467.2012*m*/*z*
41	HMDB0014700	C12012	Tigecycline	7.66_566.2610*m*/*z*
42	HMDB0014878	C07937	Riluzole	18.77_254.9817*m*/*z*
43	HMDB0014965	C08052	Cinoxacin	1.07_283.0336*m*/*z*
44	HMDB0015090	C07657	Netilmicin	8.19_475.3029n
45	HMDB0015107	C07768	Azelastine	8.86_380.1543*m*/*z*
46	HMDB0015150	C07315	Sulfamethoxazole	8.12_565.1193*m*/*z*
47	HMDB0015228	C07341	Oxamniquine	9.45_617.3309*m*/*z*
48	HMDB0015232	C07566	Pimozide	13.54_981.4670*m*/*z*
49	HMDB0015383	C07543	Ergonovine	7.36_370.1516*m*/*z*
50	HMDB0029215	C19157	Thorium	7.50_523.0885*m*/*z*
51	HMDB0029706	C19180	2-Amino-3-methylimidazo [4,5-f]quinoline	18.38_179.0734*m*/*z*
52	HMDB0030358	C09041	Aspidospermatine	10.98_359.1728*m*/*z*
53	HMDB0032287	C01913	Ferrocyanide salts	18.66_232.9280*m*/*z*
54	HMDB0033122	C10688	Glycosminine	0.88_273.0425*m*/*z*
55	HMDB0035041	C11865	Gibberellin A51	9.41_297.1492*m*/*z*
56	HMDB0035798	C09711	Piperdial	1.38_231.1381*m*/*z*
57	HMDB0037790	C15588	Polyethylene glycol	4.32_210.1106*m*/*z*
58	HMDB0040851	C08964	Calenduloside E	10.86_613.3715*m*/*z*
59	HMDB0040867	C10373	5-O-Methylembelin	19.02_309.2065*m*/*z*
60	HMDB0060669	C16584	p-Hydroxyfelbamate	7.85_567.1954*m*/*z*
61	HMDB0060676	C07493	10-Hydroxycarbazepine	0.91_291.0529*m*/*z*

**Table A4:** Common targets of PSP bioactive components across disease.

Number	Gene	Number	Gene	Number	Gene	Number	Gene	Number	Gene
1	PPARG	24	MTRR	47	CA1	70	POLE	93	ITPK1
2	MT-CYB	25	MGAT1	48	CA5B	71	CA3	94	RAD51
3	KCNH2	26	TERT	49	CA13	72	PLA2G2A	95	OAT
4	NOS3	27	STAT1	50	CA7	73	KLK1	96	PDXK
5	MMP9	28	AZGP1	51	MMP12	74	PTGS1	97	REST
6	VEGFA	29	PGF	52	MAPT	75	GABRA1	98	CD1A
7	ESR1	30	COL18A1	53	KDR	76	CAMK2A	99	ELAVL3
8	IAPP	31	IDH1	54	ALOX15	77	HSD17B8	100	MT-CO1
9	FLT1	32	ARG1	55	MMP14	78	KCNE1	101	TOP1
10	FGFR1	33	DNMT1	56	MSH6	79	SQLE	102	DNM1
11	HIF1A	34	ZPR1	57	YWHAG	80	CYP1B1	103	POLB
12	PTGS2	35	GOT1	58	CKM	81	OGT	104	MTHFD2
13	HMOX1	36	ABCB1	59	CA9	82	GABRB2	105	PCMT1
14	FABP1	37	APP	60	KIT	83	CRYM	106	DGKZ
15	EGFR	38	ALPL	61	BACE1	84	PYGM	107	MS4A1
16	BCL2	39	MET	62	CA12	85	MB	108	GRK6
17	ERBB2	40	MMP13	63	RBP3	86	TYMS	109	VAV1
18	MMP2	41	MAPK14	64	PGD	87	GABRG2	110	AGL
19	SRC	42	CES1	65	CCL24	88	UPF1	111	GLB1
20	CBS	43	GRB14	66	HADHB	89	PLA2G10	112	PLA2G5
21	HADHA	44	CA2	67	CYCS	90	PTPRJ	113	RNASE2
22	ESR2	45	LTF	68	CA6	91	WARS1	114	TBXA2R
23	FABP3	46	CA4	69	CA5A	92	GM2A		
